# Upconverting NIR Photons for Bioimaging

**DOI:** 10.3390/nano5042148

**Published:** 2015-12-04

**Authors:** Zhanjun Li, Yuanwei Zhang, Hieu La, Richard Zhu, Ghida El-Banna, Yuzou Wei, Gang Han

**Affiliations:** Department of Biochemistry and Molecular Pharmacology, University of Massachusetts Medical School, Worcester, MA 01605, USA; E-Mails: zhanjun.li@umassmed.edu (Z.L.); yuanwei.zhang@umassmed.edu (Y.Z.); hla@umassmed.edu (H.L.); zzrichard123@gmail.com (R.Z.); ghida.elbanna@gmail.com (G.E.-B.); ywei22@jhu.edu (Y.W.)

**Keywords:** upconversion nanoparticles, optical imaging, optical therapy

## Abstract

Lanthanide-doped upconverting nanoparticles (UCNPs) possess unique anti-Stokes optical properties, in which low energy near-infrared (NIR) photons can be converted into high energy UV, visible, shorter NIR emission via multiphoton upconversion processes. Due to the rapid development of synthesis chemistry, lanthanide-doped UCNPs can be fabricated with narrow distribution and tunable multi-color optical properties. These unique attributes grant them unique NIR-driven imaging/drug delivery/therapeutic applications, especially in the cases of deep tissue environments. In this brief review, we introduce both the basic concepts of and recent progress with UCNPs in material engineering and theranostic applications in imaging, molecular delivery, and tumor therapeutics. The aim of this brief review is to address the most typical progress in basic mechanism, material design as bioimaging tools.

## 1. Introduction

Upconversion is a nonlinear optical process in which long wavelength (usually near-infrared) photons are absorbed by upconversion materials which then emit photons with shorter wavelength (typically visible) [1]. Lanthanide-doped upconverting nanoparticles (UCNPs) can be excited with deep tissue penetrating NIR light (800 nm, 925 nm, 980 nm) and emit light in a broad range from ultraviolet (UV) to near-infrared (NIR) with various distinctive characteristics, such as narrow emission band, large anti-Stokes shift, and less light scattering. These attributes make them unique optical tools for biological studies [2]. In addition, these nanoparticles are non-blinking, non-photobleaching, extremely stable, and dodged the endogenous cellular fluorophores spectral window [3,4]. Thus low auto-fluorescent background can be obtained and therapeutic tracking can be conducted both *in vitro* and *in vivo*.

The working window at both NIR excitation and NIR emission (NIR-to-NIR), which are both within the biological NIR optical transmission window (700–1000 nm), are particularly important for *in vivo* imaging of small animals, because it permits deep tissue penetration and less absorption and scattering of biotissues and organs [5]. The unique advantages of UCNPs make them an ideal nanoplatform for diverse applications in both imaging and therapy: (i) They can serve as background-free optical probes for targeted imaging [6]. (ii) They can be adapted as traceable nanocarriers to precisely deliver therapeutic cargoes, in which way the side effects are minimized in nearby tissues and organs [7]. (iii) UCNPs can be used as remote control photoswitches to precisely control the therapeutic procedures in deep tissue [8].

In this brief review, we included the most typical and important progresses in UCNPs to acknowledge the basic mechanisms, chemical and physical attributes, bio-medical and imaging applications of UCNPs. The most recent progress of utilizing UCNPs as bioimaging nano-platform and phototherapeutic reagents are particularly emphasized.

## 2. Engineering of UCNPs for Biomedical Applications

### 2.1. Basic Mechanism of UCNPs

**Figure 1:**
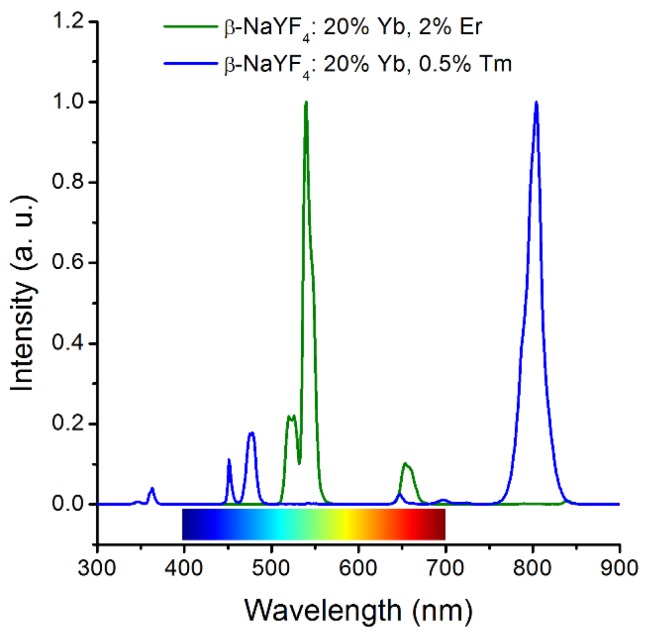
Typical upconversion spectra of β-NaYF_4_:20%Yb, 2%Er (dark green) and β-NaYF_4_:20%Yb, 0.5%Tm (blue).

**Figure 2:**
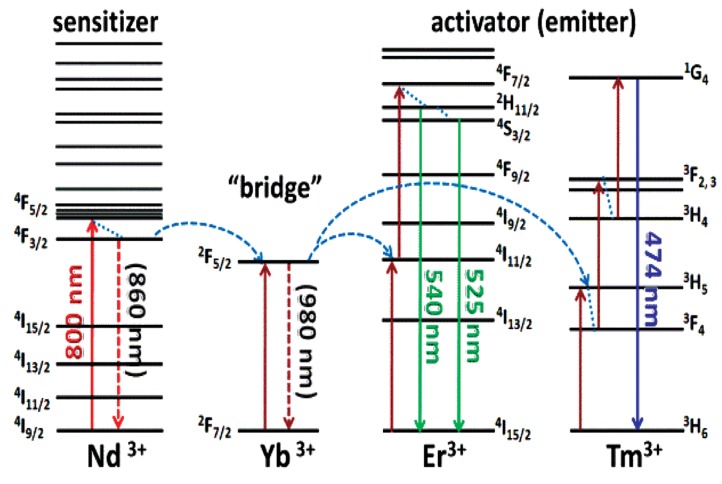
Upconversion process of Nd/Yb/Er(Tm) tri-dopants system with 800 nm excitation. Reproduced with permission from [12]. Copyright John Wiley & Sons, 2013.

In general, lanthanide-doped UCNPs contains three essential components, (i) sensitizer (usually Yb^3+^), (ii) emitter (usually Tm^3+^, Er^3+^, Ho^3+^), (iii) host matrix. Because of the requirements in the matching of energy levels, the most effective and popular sensitizer/emitter pairs are found to be Yb/Er, Yb/Tm (Figure 1). The upconversion host matrix must have low phonon energies, thus lattice stress and non-radiative pathways can be minimized. In general, hexagonal AReF_4_ (e.g., A = Li/Na/K, Re = Y/Lu/Gd) is broadly considered as the best host matrix for UCNPs [9,10,11]. Recently, we found that Nd^3+^, which has a strong absorption at ~800 nm, can be used as a novel sensitizer (Figure 2) [12]. By the fabrication of a tri-doped NaYF_4_:Nd, Yb, Er/Tm, the excitation wavelength of lanthanide-based UCNPs can be transferred to 800 nm. This design not only offers a new excitation choice, but also can decrease the thermal effect obviously because the absorption coefficient of water at 800 nm is several magnitudes lower than that at 980 nm. Thus, we can minimize the thermal side effects during upconversion imaging or therapy by using these tri-doped UCNPs. It should be noted that the upconversion efficiency of this simple tri-doped system is low because of the reverse energy transfer from Er/Tm to Nd. Soon after our report, this problem was solved by fabrication of a core@shell structure which will be discussed later.

### 2.2. Synthesis of UCNPs

Due to the requirement of narrow size distribution and high dispersibility for biological studies, thermal decomposition and solvothermal synthesis techniques are the most widely employed strategies. In addition, UCNPs can be fabricated with diverse sizes and shapes in controlled conditions, such as temperature, materials ratio, and selected component [13]. In order to generate solvent dispersible UCNPs, Yan *et al.* developed a high temperature decomposition strategy, and a variety of monodispersed cubic and hexagonal NaYF_4_ based nanocrystals were fabricated with high quality [14]. Generally, the size and morphology of UCNPs can be well controlled by decomposing metal trifluoroactate precursors at high temperature in mixed solvents of oleic acid, oleylamine and octadecene. This strategy has become the most typical method to fabricate UCNPs, and the critical synthesis parameters have been thoroughly investigated, such as coordinating solvent composition [15], decomposition temperature [16], starting material species and ratio [17], and core/shell structures [18]. For example, to synthesize high luminescent and ultra-small upconversion nanoparticles with biomolecules comparable size, Chow *et al.* fabricated *ca.* 11 nm -NaYF_4_:Yb,Er and -NaYF_4_:Yb,Tm UCNPs in pure oleylamine solutions, and further upconversion efficiency was improved by coating inert shell structure [15].

Using hydro-thermal synthesis strategy, UCNPs with uniform sizes and shapes can also be achieved. Generally, UCNPs are obtained by mixing fluoride salts (e.g., NH_4_F) with lanthanide compounds in solvents with high boiling points such as ethylene glycol, and reacted under high temperature and pressure. For example, using this method, β-phased NaYF_4_ UCNPs was fabricated with corresponding oleate precursor [19]. A more sophisticated strategy was developed using a liquid-solid-solution phase-transfer method in water/alcohol/oleic acid mixture to fulfill predictable size, shape and phase UCNPs [20,21].

### 2.3. UCNPs with Core@Shell Structures

**Figure 3:**
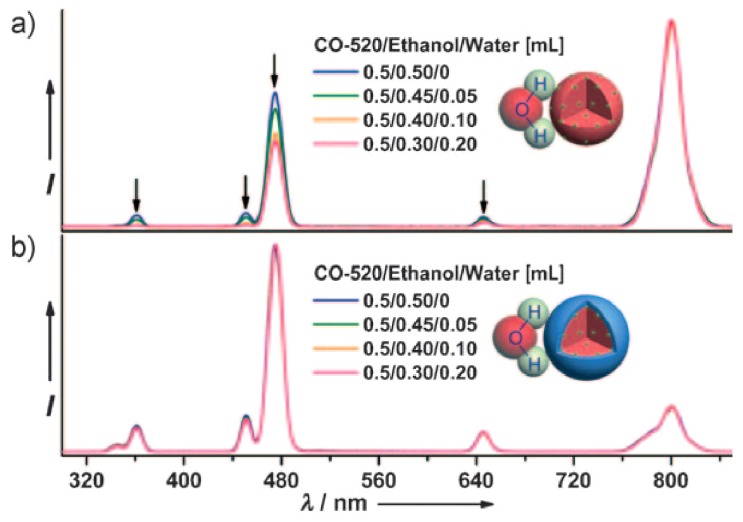
Upconversion emission spetra of (**a**) NaGdF_4_:25%Yb/0.3%Tm (15 nm) and (**b**) corresponding core-shell nanoparticles (20 nm) in nonylphenylether/ethanol/water solutions with different water ratio. Reproduced with permission from [23]. Copyright John Wiley & Sons, 2010.

As optical bio-probes, high fluorescent efficiency will be required in water solutions. However, the OH and NH_2_ groups surrounding the surface of UCNPs could quench the excited states through nonradiative relaxation processes. Nevertheless, when the nanoparticles sizes get smaller, those quenching effects become more severe. In order to conquer this problem, an inert shell (undoped NaYF_4_, NaGaF_4_, NaLuF_4_, *etc.*) is usually needed to protect the upconverting active core (namely a core@shell nanostructure) from the outside quenching reagents. Thus the possibility of energy loss from the active core is highly reduced and consequently enhancing upconversion efficiency. For example, Kong and Zhang carefully studied the β-NaYF_4_:Yb^3+^,Er^3+^@β-NaYF_4_ core-shell structure. From their kinetics analysis, they discovered the quenching effect of luminescent centers can be effectively reduced by homogeneous coating with NaYF_4_ shell, and enhance luminescence [22]. Later on, Liu *et al.* showed direct evidence for the surface quenching effect related to the nanoparticle sizes in NaGaF_4_:Yb,Tm. They also intensified the optical integrity and showed surface quenching effects of the nanoparticles can be greatly preserved after an inert thin shell coating (NaGdF_4_:Yb,Tm@NaGdF_4_) (Figure 3) [23].

**Figure 4:**
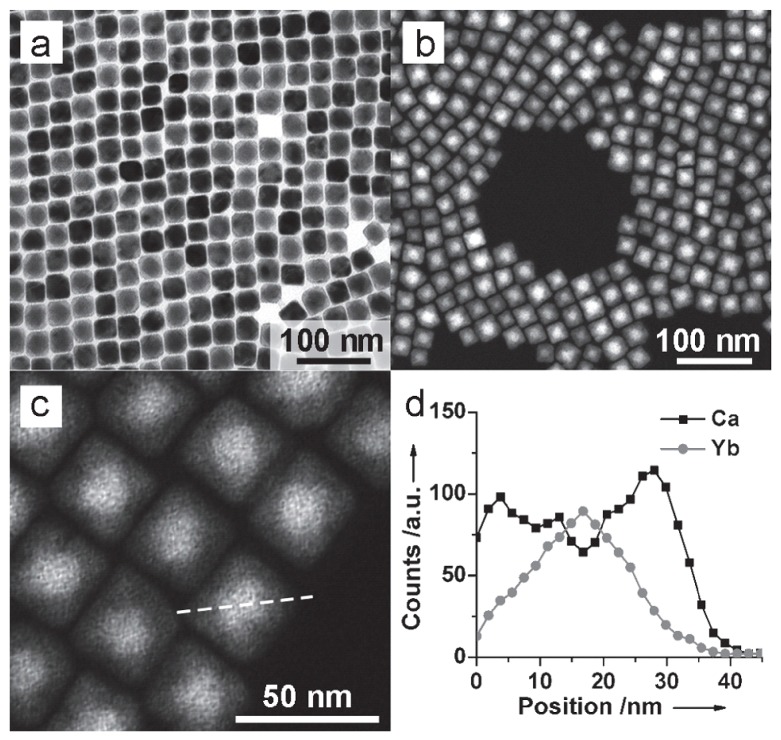
Heteroshell structure of α-NaYbF_4_:Tm@CaF_2_. (**a**)TEM, (**b**) high-angle annular dark-field STEM, (**c**) linear EDX scanning of a single UCNP and (**d**) corresponding elemental ratio analysis. Reproduced with permission from [24]. Copyright John Wiley & Sons, 2013.

Usually, β-NaY/Gd/LuF_4_ is considered to be the best upconversion matrix. The same host matrix will be used in the active core@inert shell design. Quite interestingly, we find that an epitaxial CaF_2_ heteroshell can increase the upconversion efficiency of α-NaYF_4_ by a hundred-fold (Figure 4). The UV upconversion in α-NaYbF_4_:Tm@CaF_2_ (362 nm) is even higher than β-NaYF_4_:30%Yb, 0.5%Tm@β-NaYF_4_ and larger β-phase counterparts [24]. In addition, the CaF_2_ coating is found to be more effective in resisting quenching in aqueous medium to preserve the upconverting UV emissions. In the same material, we also found that CaF_2_ heteroshell could increase the NIR emission of α-NaYbF_4_:0.5%Tm for 35 times with a relative high NIR-to-NIR quantum yield of 0.6% under low energy excitation of 0.3 W/cm^2^ [5].

**Figure 5:**
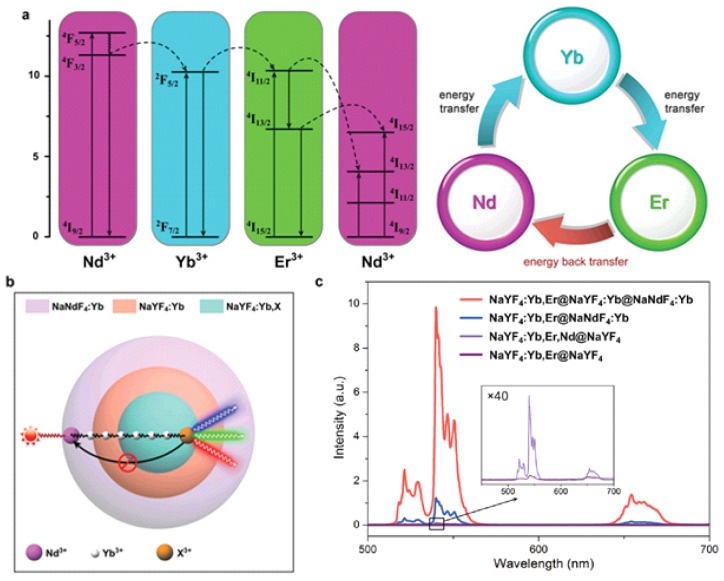
Bright upconversion under 800 nm excitation by engineering core@shell@shell structure. (**a**) Simplied energy-level diagrams depicting the energy transfer between Nd, Yb, and Er ions upon 800 nm excitation. (**b**) Schematic illustration of the proposed energy transfer mechanisms in the quenching-shield sandwich-structured UCNPs, (**c**) upconversion emission spectra of the as-synthesized UCNPs. Reproduced with permission from [25]. Copyright John Wiley & Sons, 2014.

For the Nd^3+^ sensitized UCNPs, the upconversion efficiency using 800 nm excitation is relatively lower than the traditional 980 nm excited counterparts. In order to address this issue, Zhao and Yao fabricated a quenching-shielded sandwich-structured rare-earth nanoparticles that have high upconversion emissions with excitation at wavelength of 800 nm, as shown in Figure 5 [25]. In this system, the nanostructure has been well-defined to eliminate the potential cross-relaxation pathways between the activator and sensitizer by introducing an interlayer of NaYF_4_:Yb^3+^. The emission intensity of the nanoparticles reached maximum when the interlayer thickness was ~1.45 nm (NaYF_4_:Yb^3+^). This well-defined unique nanostructure is essential to eliminate the deleterious cross-relaxation pathways between the activator and sensitizer by means of a precisely controlled transition layer. The as-synthesized UCNPs are even brighter than conventional 980 nm-excited nanoparticles at low excitation power density of 0.5 W/cm^2^. Furthermore, the use of an 800 nm laser source instead of a 980 nm one can increase the penetration depth in biotissue and suppress overheating. It is important to note that according to the American National Standard for Safe Use of Lasers, the maximum permissible light exposure for skin of human beings at 800 nm is calculated to be lower (0.32 W/cm^2^) than that at 980 nm (0.73 W/cm^2^) [26]. Thus one should pay special attention to the power density of 800 nm for *in vivo* research.

**Figure 6:**
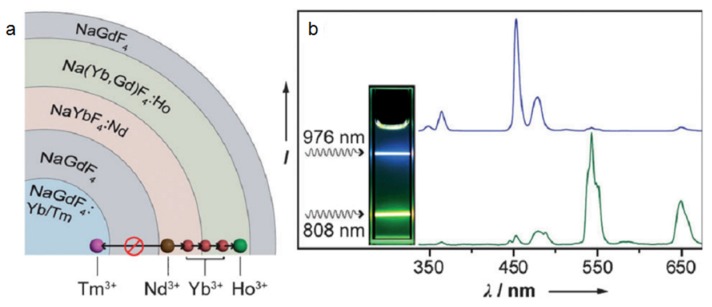
(**a**) Schematic design of tuning the Nd-sensitized upconversion process through nanostructural engineering. (**b**) Emisssion spectra of the multishelled nanoparticles under excitation at 808 and 976 nm, respectively. Inset: digital camera photograph of corresponding solution sample. Reproduced with permission from [27]. Copyright John Wiley & Sons, 2013.

By simultaneously harnessing the sensitizer Yb^3+^ and Nd^3+^ together into different shell layers of one single nanoparticle, Wang *et al.* designed a kind of core@multishell nanostructure named NaGdF_4_:Yb,Tm @NaGdF_4_ @NaYbF_4_:Nd @Na(Yb,Gd)F_4_:Ho @NaGdF_4_, in which different emissions can be realized by altering excitation light source (976 nm/blue, 808 nm/green, Figure 6) [27]. Under 976 nm laser excitation, the excitation light will absorbed by Yb^3+^ in both the core and the shell of the UCNPs. However, the energy back transfer from Ho^3+^ to Nd^3+^ will inhibit the emission of Ho^3+^ (green). The general emission color will be dominated by transition from Yb to Tm in the core. But, under 808 nm excitation, Nd^3+^ will be sensitized and then transfer the absorbed energy to Ho^3+^ to generate green emission. The energy transfer to the inner core is blocked by the inert NaGdF_4_ layer between the Tm^3+^ activated core and the Ho activated shell. Thus the 808 nm excited emission will be dominated by Ho^3+^ (green). This kind of excitation-regulated upconversion emission properties will have great potential in the field of multichannel photoactive processes.

**Figure 7:**
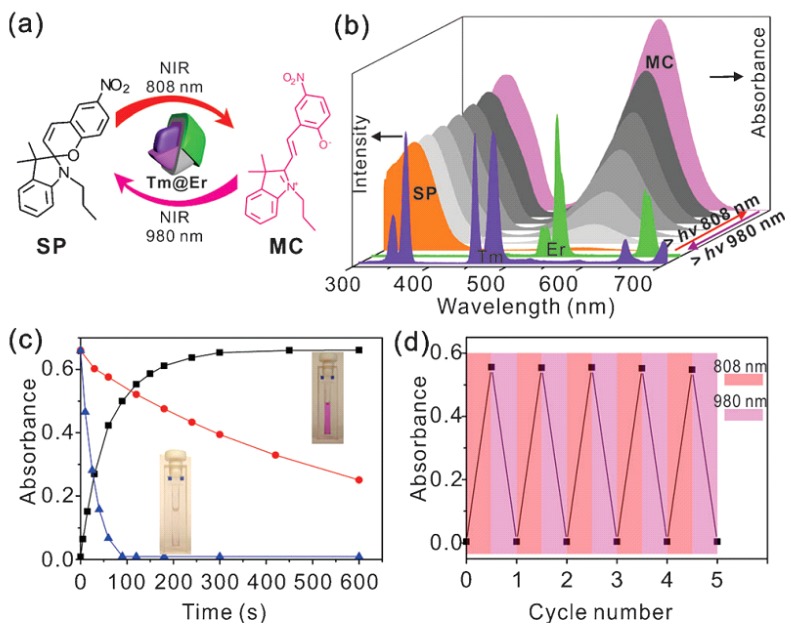
(**a**) Illustration of two-way photoswitching of spiropyran by using UCNPs with dual NIR excitations. (**b**) Tm^3+^ and Er^3+^ emissions from the UCNPs under 808 nm and 980 nm excitations and the evolution of the UV-vis absorption spectrum of the photoisomerization. (**c**) Kinetic monitoring of the photoswitching reaction. The red line shows the kinetics of the reaction of merocyanine to spiropyran. (**d**) Dual NIR-driven photoswitching of spiropyran over many cycles in THF/methanol (9/1, *v*/*v*) solution by monitoring the absorbance of merocyanine at 560 nm. Reproduced with permission from [28]. Copyright John Wiley & Sons, 2014.

Lee’s group synthesized a similar dual-channel upconversion system and more importantly they demonstrated the two-way photoswitching applications of this novel UCNPs (Figure 7) [28]. The photoisomerization between spiropyran and merocyanine can be regulated by UV and visible light. The maximum absorption peaks of spiropyran and merocyanine that locate at 342 nm and 560 nm overlap well with the UV emission from the Tm^3+^ and Er^3+^ respectively. Thus, under excitation of 808 nm, UV emission will be activated by energy transfer from Nd^3+^ to Tm^3+^, leading to the photoisomerization of spiropyran to merocyanine (pink solution). Thereafter, under the excitation of 980 nm laser, green emission from Er^3+^ can be generated, leading to the photoisomerization of merocyanine back to spiropyran (colorless).

### 2.4. Surface Modification for Upconversion Enhancing and Bio-Conjugation

Post-modification of the surface of UCNPs by charged or polar groups can offer them aqueous solubilities and bio-compatibilities. In this regard, several strategies have been developed to transfer the as-synthezied hydrophobic nanoparticles into water solution using amphiphilic polymer [29], and ligand oxidation [30]. Firstly, Yadong Li’s group developed a polyol solvent ligand exhange method to transfer hydrophobic inorganic nanocrystals from organic solvent to aqueous solution by modifying polyelectrolytes, such as poly(acrylic acid), poly(allylamine) and poly(sodium styrene sulfonate) [31]. In this method, hydrophobic nanoparticles in toluene are injected rapidly into a heated solution of polyol (diethylene glycol) and exchanging ligands. The solution becomes turbid immediately because of the insolubility of hydrophobic nanocrystals in the polar solvent. Upon continued heating at a higher temperature close to the boiling point of the solvent, the solution slowly turns clear, indicating the occurrence of ligand exchange and dissolution of nanocrystals in DEG. Adding excessive hydrochloric acid can then precipitate the nanocrystals. The resulting products can then be redispersed in aqueous solution. This method is found also effective in the case of UCNPs.

**Figure 8:**
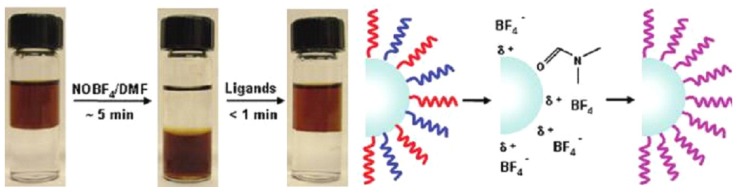
Scheme of the phase transition and ligand exchange procedure by using NOBF_4_. Reprinted with the permission with permission from [32]. Copyright American Chemical Society, 2011.

Another effective phase transition method was developed by Dong *et al.* characterized by using NOBF_4_ (Figure 8) [32]. In this method, nitrosonium tetrafluoroborate (NOBF_4_) is utilized to remove the organic ligands on the nanoparticle surface, stabilizing the nanoparticles in various polar, hydrophilic media such as *N,N*-dimethylformamide for long term storage. The afforded hydrophilic nanoparticles can be further tailored via diverse types of functional groups. Furthermore, the phase transition and ligand exchange reaction is so fast that it can be finished in several minutes. And more significantly, the hydrophilic nanoparticles obtained by NOBF_4_ treatment can readily undergo secondary surface modification due to the weak binding affinity of BF_4_^−^ anions to the particle surface, allowing reversible phase transition. Regarding the application of UCNPs, this method tends to be widely accepted as a facile phase transfer process because of the ultrafast phase transfer speed, moderate reaction situation, and simple operation [5,12,29,33,34].

## 3. UCNPs as Imaging Contrast Reagents

**Figure 9:**
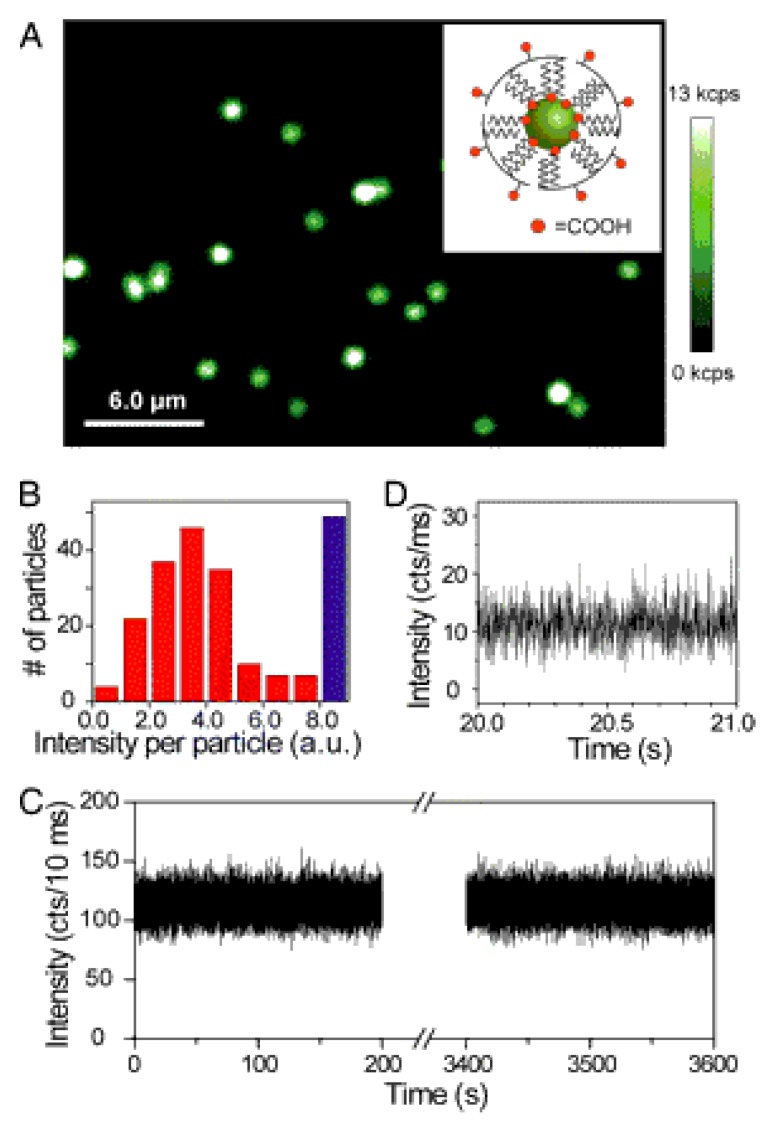
Upconverted luminescence of individual water-soluble upconverting nanoparticles (UCNPs). (**A**) Confocal upconverted luminescent image of individual amphiphilic polymer-coated UCNPs (schematically shown in the *Inset*) sparsely dispersed on a clean coverglass. The laser power is approximately 10 mW, equivalent to approximately 5 × 10^6^ W/cm^2^. Some of the bright luminescent spots represent multiple UCNPs within the diffraction limited area, generating saturated “white” spots in the image. (**B**) A histogram of integrated emission intensity from over 200 upconverted luminescent spots, suggesting that most of the luminescent spots are from single polymer-coated UCNPs. The data were analyzed from confocal upconverted luminescent images over a 75 × 75 μm area, and the number of saturated “white” spots was shown in the histogram as a blue bar. Such single water-soluble UCNPs also exhibit exceptional photostability (**C**) and non-blinking behavior (**D**) Reprinted with the permission from [3]. Copyright American Chemical Society, 2009.

**Figure 10:**
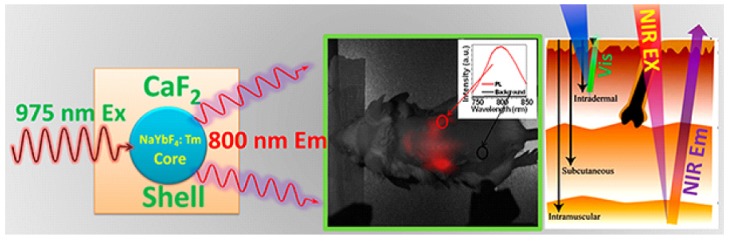
Example of lanthanide-doped UCNPs of core-shell structures with NIR-to-NIR optical transitions and their application for small animal imaging studies plus illustration showing the better penetration of NIR light in contrast with visible light. Reprinted with permission from [5]. Copyright American Chemical Society, 2012.

The upconversion property of UCNPs makes them very appealing for bio-imaging studies due to their zero autofluorescence derived from biological samples. The near-infrared excitation wavelength also permits deep tissue penetrations. In addition to the non-blinking and stable properties, UCNPs can conduct improved detection limits with large signal-to-noise ratio compared with classical imaging probes, like organic dyes and quantum dots. For example, β-NaYF_4_:Er,Yb nanocrystals were fabricated for luminescence imaging studies, the nanoparticles with size around 30 nm was found to have improved signal-to-noise ratio, and to be non-photobleaching and non-blinking (Figure 9) [3]. After encapsulating with amphiphilic polymer, the UCNPs can be dispersed in water solutions, and further incubated with murine fibroblasts and inoculated by endocytosis. In another example, NaYbF_4_-based UCNPs have been synthesized with CaF_2_ shell coating. Both *in vitro* and *in vivo* imaging were performed with high contrast imaging properties after tail vein injection to mouse, demonstrating the efficiency for practical imaging researches [5] (Figure 10).

**Figure 11:**
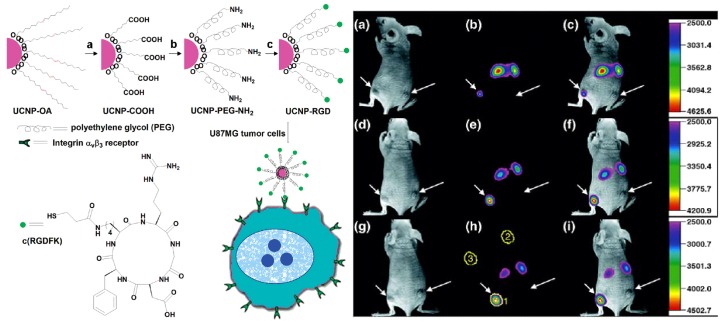
Illustration scheme for UCNP-RGD and *in vivo* upconversion luminescence imaging of subcutaneous U87MG tumor (left hind leg) and MCF-7 tumor (right hind leg) after intravenous injection of UCNP-RGD conjugate over 24-hour period. (**a**,**d**,**g**) bright field, (**b**,**e**,**h**) upconversion images, (**c**,**f**,**i**) overlay of the corresponding bright field images with the upconversion ones. (**a**–**c**), (**d**–**f**), and (**g**–**i**) are taken at 1, 4 and 24 h postinjection, respectively. Reprinted with permission from [35]. Copyright American Chemical Society, 2009.

Because many tumor cells overexpress corresponding transmembrane receptors, UCNPs with small peptide-labeling are particularly interesting for tumor-targeting drug delivery tracking within complex biological systems. For example, peptide motif of c(RGD) with high binding affinity to α*_v_*β_3_ integrin receptor was used to functionalize UCNPs for *ex vivo* and *in vivo* imaging studies. Intense luminescence from the tumor could be observed 24 h after injection, while luminescence from the liver was reduced significantly (Figure 11). Also, due to the specific peptide labeling, the afforded nano-system was found to have more affinity to U87MG tumor cell line compared with MCF-7 tumor, suggesting a potential application as a cancer detection technique [35]. Another polypeptide, neurotoxins, was shown to effectively bind with many types of cancer cells of high specificity and affinity. For instance, a typical neurotoxin peptide of recombinant chlorotoxin was used to conjugate with hexagonal-phase NaYF_4_:Yb,Er/Ce nanoparticles, which were later injected into Balb/c nude mice [36]. The sensitivity and specificity of neurotoxin-mediated UCNPs tumor targeting was readily imaged using 980 nm excitation.

## 4. UCNPs as Imaging Guidable Delivery Nanoplatform

### 4.1. UCNPs as in Vivo Traceable Drug Carriers

Using drug delivery carriers can greatly enhance the efficacy of pharmaceutical payloads for the improved solubility, stability, and pharmacokinetics of drugs. A wide variety of materials have been used as drug carriers, and more recently, fluorescent quantum dots have been used as platforms of optical imaging-guided carriers in real time in live organisms. However, the highly toxic components in quantum dots limited its further applications for studies in cells and small animals. UCNPs systems with unique optical and biocompatible components have emerged as promising candidates for traceable drug delivery.

**Figure 12:**
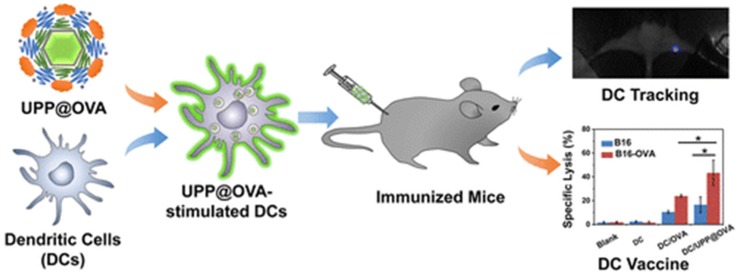
Schematic illustration of antigen-loaded UCNPs for dendritic cell stimulation, tracking, and vaccination in immunotherapy. Reprinted with permission from [39]. Copyright American Chemical Society, 2015.

Two of the most important UCNP based drug delivery systems are (i) UCNP@porous silica core-shell structure [33,37] and (ii) drug conjugated or absorbed UCNPs. In the first method, drugs can be deposited in the pores of mesoporous silica shell coated onto UCNPs. Ibuprofen was used as a model drug, and loaded onto mesoporous silica-coated-NaYF_4_:Yb,Er UCNPs fibers by electro-spinning process [38]. Large ibuprofen loading amounts can be performed because of the high specific surface area and large pore volume of silica shell. After loading ibuprofen, significant upconverting luminescence quenching will occur, thus the loading quantity can be determined by measuring the quenching extent, more importantly, the subsequent drug releasing can be monitored by the recovering of luminescence intensity. On the other hand, in a recent collaborative report lead by Liu’s group, antigen-loaded UCNPs are used to label and stimulate dendritic cells, which could be traced upon being administered into animals and trigger an antigen-specific immune response (Figure 12) [39]. It has been discovered that a model antigen, ovalbumin, was able to conjugate on the surface of di-polymer-coated UCNPs through electrostatic interaction, which induces nanoparticle antigen complexes and are efficiently engulfed by dendritic cells and induce their maturation and cytokine release. Highly sensitive *in vivo* upconversion luminescence imaging of nanoparticle-labeled dendritic cells is achieved. Dendritic cells were observed to target draining lymph nodes thereafter. In addition, strong antigen-specific immune responses including enhanced T cell proliferation, interferon gamma (IFN-γ) production, and cytotoxic T lymphocyte mediated responses are induced by a nanoparticle-pulsed dendritic cell vaccine, which is promising for dendritic cell-based immunotherapy potentially against cancer.

### 4.2. Light Controllable Drug Release Based on UCNPs

**Figure 13:**
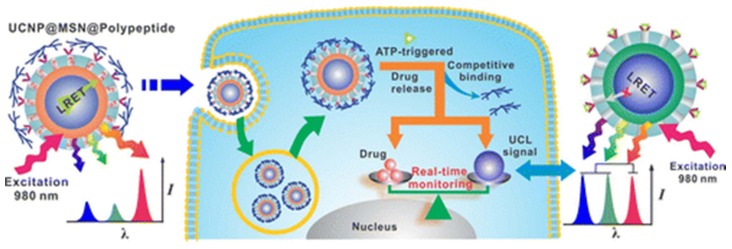
Scheme of real-time monitoring of ATP-responsive drug release using mesoporous-silica-coated multicolor upconversion nanoparticles. Reprinted with permission from [41]. Copyright American Chemical Society, 2015.

Releasing bioactive molecules or drugs at desired time and location is of great interest for delivery systems development, in which way determinative therapeutic and diagnostic consequence can be greatly enhanced with minimized side effects during treatment. In addition, studies at directing cellular processes and disease regulation would benefit from the delivery of multi-payloads in varied doses at different time intervals [34,40]. Among the release controlling methods, light-triggered molecular cleavage has received growing interest, due to the well-defined time and spatial drug releasing, thus offering unique precisely compared with other classical stimuli. Using the upconversion properties of UCNPs, remote control of photo-release of imaging probes and drug payloads have been demonstrated. For example, NIR light-responsive crosslinked mesoporous silica coated UCNPs drug delivery conjugate has been designed as photocaged nanocarriers [40]. The lanthanide doped UCNPs was first coated with silica shell, and followed by polymerization of 1-(2-nitrophenyl)ethyl photocaged oligo(ethylene) glycol vinyl monomers. Antitumor drug (doxorubicin) was able to be encapsulated in the hydrophobic pockets within the aforementioned polymer. Under NIR light irradiation, the cleavage of the crosslinked photocaged linker can be triggered by the upconverting emissions from UCNPs and precisely release of targeted drug can be performed at controlled position and regulated time. Moreover, the multi-wavelength emission of UCNPs can be harnessed to monitor the real time drug release based on ratiometric fluorescence assay. In this regard, Lee’s group developed a polypeptide-wrapped mesoporous-silica-coated multicolor upconversion nanoparticle (UCNP@MSN) as an adenosine triphosphate (ATP)-responsive drug delivery system (DDS) for long-term tracking and real-time monitoring of drug release, as shown in Figure 13 [41]. Their UCNP@MSN with multiple emission peaks in UV-NIR wavelength range was functionalized with zinc dipicolylamineanalogue (TDPA-Zn^2+^) on its exterior surface and loaded with small-molecule drugs like chemotherapeutics in interior mesopores. Due to multivalent interactions between Asp moieties of the polypeptide and the TDPA-Zn^2+^ on the UCNP-MSNs, these drugs were kept within the UCNP-MSNs when the nanoparticles were coated by a branched polypeptide, poly(Asp-Lys)-b-Asp. This led to luminescence resonance energy transfer (LRET) from the UCNPs to the entrapped drugs, which typically have absorption in UV-visible range, ultimately resulting in quenching of UCNP emission in UV-visible range while retaining their strong NIR emission. Because of its higher affinity to TDPA-Zn^2+^, addition of ATP led to a competitive replacement of the polypeptide by ATP that resulted in the release of the drugs and concomitant elimination of LRET. The release of the entrapped drugs can be monitored in real-time by harnessing such ATP-triggered ratiometric changes in LRET. The proposed UCNP@MSN-polypeptide hybrid nanoparticle has great potential for stimuli-responsive drug delivery as well as for monitoring biochemical changes taking place in live cancer and stem cells.

### 4.3. UCNPs for Gene Delivery

**Figure 14:**
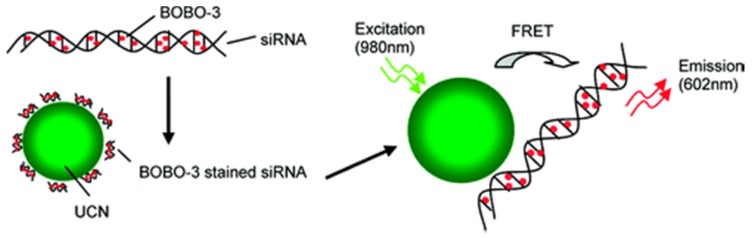
Schematic drawing of FRET-based UCNPs/siRNA-BOBO-3 complex system. siRNA are stained with BOBO-3 dyes, and the stained siRNA are attached to the surface of NaYF_4_:Yb,Er nanoparticles. Upon excitation of the nanoparticles at 980 nm, energy is transferred from the donor (UCNPs) to the acceptor (BOBO-3). Reprinted with permission from [42]. Copyright American Chemical Society, 2010.

The limitation for gene therapy is designing effective gene delivery vectors for specific and controllable delivering gene cargoes into living cells and tissues. There is a growing interest for developing non-viral synthetic nano-carriers for gene delivery. Such carriers require four important factors, namely that they are (i) biocompatible, (ii) traceable by long-term and real-time imaging, (iii) controllable release, and (iv) targeted. UCNPs have been employed to deliver nucleic acids in gene therapy, and exhibit promising benefits. For example, silica-coated -NaYF_4_:Yb, Er UCNPs was used to track the delivery of siRNA into cells using luminescence resonance energy transfer system in order to explore the parameters within live cells such as intracellular uptake, and biological stability of UCNPs-bound siRNA [42]. In this system, cationic silica-coated -NaYF_4_:Yb, Er act as the donor and the siRNA-intercalating dye BOBO-3 as the corresponding acceptor. Under 980 nm excitation, the nanoparticles emit upconverted fluorescence at the wavelength of 543 nm, which was consequently absorbed by BOBO-3-stained siRNA (siRNA-BOBO-3) acceptor via overlapped absorption band. This UCNP was demonstrated to bind and protect siRNA from RNase cleavage during the delivery of siRNA into cells. When the amino-modified UCNPs was bound with BOBO-3-stained siRNA, with 980 nm laser excitation, energy was allowed to transfer from UCNPs to BOBO-3, and consequently induce BOBO emissions at the wavelength of 602 nm (Figure 14). Once the siRNA was separated from UCNPs, the energy transfer process was inhibited. The results showed that siRNA could gradually release from the UCNPs surface into the cytoplasm over 24 h. Subsequently, similar strategy was used to monitor green fluorescent protein (GFP)-encoded plasmid DNA delivery and release in live cells [43].

## 5. UCNPs as Phototherapeutic Reagents

### 5.1. Photodynamic Therapy

Typical photosensitizer is capable of excitation under visible light and generates cytotoxic reactive oxygen species (ROS) which can be used to kill tumor cells. Only light is used as the therapeutic reagents in such a process, namely photodynamic therapy (PDT) [44]. Due to the shallow penetration depth of visible light that commonly used for the commercial available photosensitizers, conventional PDT has been limited in the superficial and flat lesions treatment. Recently, the application of deep penetrating near-infrared light excitable UCNPs in conjunction with PDT has shown to have clear potential in the treatment of solid tumors due to its ability to penetrate thick tissue [8,45,46]. The design of a UCNPs-based PDT system includes the aforementioned UCNPs, surface coating, and the hereafter photosensitizers. Ideally, a photosensitizer should have good ROS production efficiency, a high absorption coefficient at longer wavelengths for better tissue penetration, amphiphilicity, low dark toxicity, ease of synthesis and ease of formulation in aqueous solvents for *in vivo* delivery [47,48,49]. Several different kinds of photosensitizers have been developed for PDT and they can be broadly classified into chemical groups such as porphyrins, phthalocyanines, chlorins, 5-aminolevulinic acid (ALA) and naphthalocyanines, photosensitizers like porfimer sodium, temoporfin, 5-aminolevulinic acid, methyl aminolevulinate, hexyl aminolevulinate, talaporfin sodium, aluminium phthalocyanine disulphonate, tin ethyl etiopurpurin and verteporfin have been approved for clinical use [8].

**Figure 15:**
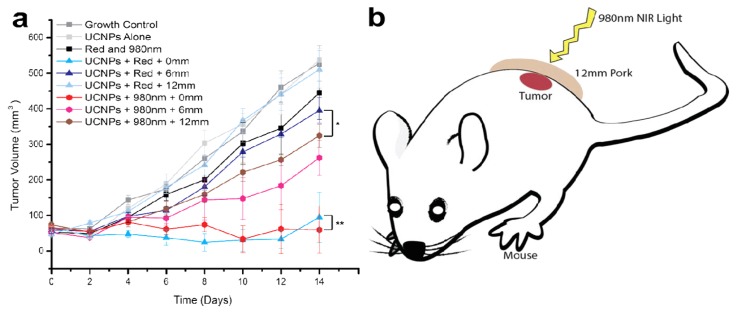
(**a**) *In vivo* volume of tumors exposed to various controls and ALA-UCNPs with red and near-infrared irradiation (0.5 W/cm^2^) in simulated deep tumors, (**b**) scheme of the simulated deep tumor PDT process. Reprinted with permission from [51]. Copyright American Chemical Society, 2014.

Recently, we developed high Yb doped UCNPs with a biocompatible CaF_2_ shell with an optimal 15-fold increase in red-emissions compared to their hexagonal phased counterparts. The absolute quantum yield of α-NaYF4:80%Yb,2%Er@CaF_2_ is measured to be 3.2% ± 0.1%, the highest reported value for red-emissions. Furthermore, we demonstrate conjugating ALA (aminolevulinic acid), a clinically used prodrug for the red absorbing photosensitizer PpIX, to the UCNP surface via a hydrazone linkage. This photodynamic therapy system was tested for its therapeutic potential: it exhibited strong singlet oxygen generation and ~70% cell death after 20 min of NIR irradiation. Furthermore, significant cell death (~30%) was produced in simulated deep tumor conditions with as much as 12 mm of pork tissue and a biocompatible low power density of 0.5 W/cm^2^ while ALA UCNPs based on the known red-emitting UCNP cannot. Finally, *in vivo* mice models of tumors when treated with these ALA UCNPs demonstrated significant size reduction from the controls even under 12 mm of pork tissue (the deepest tissue penetration depth that UCNP PDT has reached ), while red light in clinical use could not be able to achieve (Figure 15) [50]. This study marks an important progress in regard to biocompatible photodynamic therapy via UCNPs so as to treat deep-set tumors at a low laser power density. It may also provide new opportunities for a variety of applications using upconverting red radiation in photonics and biophotonics. It should also be noted that there is still no solid conclusion in regard to the maximum depth that NIR light can reach. Although some model tissue, such as the fresh bovine meat in the literature [51], the systematic research with respect to different types of organs and tissues at diverse anatomic locations in different animals is lacking. Moreover, despite much progress, the use of UCNPs for deep-tissue drug delivery or photodynamic therapy has been challenging. Thus the improvement of quantum efficiency of UCNPs is highly important in the field [52,53,54].

**Figure 16:**
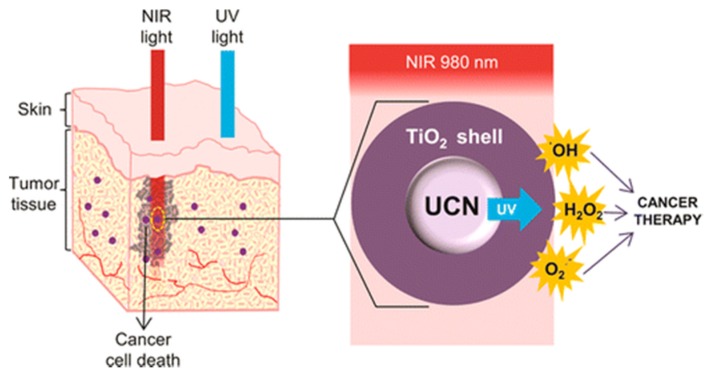
Schematic illustration of the NIR-driven reactive oxygen species generation by the use of UCNP/TiO_2_. Reprinted with permission from [55]. Copyright American Chemical Society, 2015.

For the PDT process mediated by organic photosensitizer, three common strategies are generally used to load sensitizer onto UCNPs, including (i) silica encapsulation, (ii) covalent conjuation and (iii) physical adsorption. However, none of these strategies could guarantee controlled loading/loading of sensitizer. Therefore, it becomes essential to design controllable and stable sensitizer loading, for optical and reproducible therapeutic efficiency. In this regard, almost asimultaneously, Lin’s group [55] and Zhang’s group [56] reported the design of using TiO_2_ surface coating on UCNPs as stable and reliable photosensitizer and demonstrate the ability of the synthesized nanostructure in achieving cancer cell killing both *in vitro* and *in vivo*, respectively (Figure 16). In their works, they presented a method to coat a thin layer of TiO_2_ on individual UCNP core. Our design allows controllable and highly reproducible photosensitizer loading, preventing any leakage of sensitizer compared to previously developed nanoconstructs, thus ensuring repeatable PDT results. Further surface modification of the developed nanoconstructs with polyethylene glycol (PEG) enabled them to be biocompatible, and showed excellent therapeutic effects both *in vitro* and *in vivo*.

### 5.2. Photothermal Therapy

The basic model for photothermal therapy (PTT) is in part similar with PDT, in which vibrational energy (heat) is generated from photosensitizer for cell killings [57]. β-NaYF_4_:Yb,Er nanoparticles were coated with silver shell with stronger plasmonic resonance performance, and PTT was applied *in vitro* on HepG2 and BCap-37 cells from human hepatic cancer and human breast cancer separately [58].

**Figure 17:**
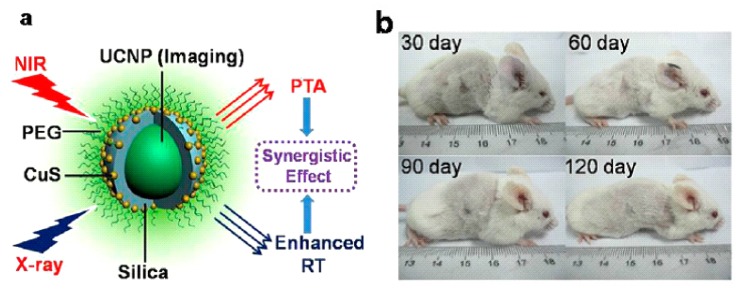
(**a**) Illustration of nano-carriers for enhanced photothermal ablation and radiotherapy synergistic therapy; (**b**) photographs of mice in 30, 60, 90 and 120 days of treatment, showing complete eradication of the tumor and no visible recurrences of the tumors in at least 120 days. Reprinted with permission from [59]. Copyright American Chemical Society, 2013.

In order to achieve a multimodal cancer therapy, multifunctional core/satellite nanotheranostic was developed by attaching ultrasmall CuS nanoparticles onto the surface of silica-coated UCNPs (Figure 17) [59]. These nano-composite exhibit many advantages as multifunctional nanotheranostics, such as (i) NIR light can be transferred to local thermal energy for photothermal therapy agents; (ii) Z elements (Yb, Gd, and Er) can cause large local radiation dose-enhancement around nanoparticles as radiosensitizers; (iii) UCL/MR/CT trimodal imagines could be simultaneously performed with this multifunctional nanotheranostic. This development laid the groundwork for the image-guided therapy in the future.

## 6. Conclusion and Prospects

In the past decade, we have witnessed the rapid development of UCNPs both in synthesis and applications. The unique optical properties and outperformed chemical and physical behaviors of UCNPs make them attractive candidates for bioimaging applications. Their UV/vis emission can be excited by bio-tissue penetrable NIR light, which endow them with deep tissue applications such as remote switchable smart bio-devices, NIR-driven PDT process. However, the upconversion efficiency of lanthanide-based UCNPs is still very low, especially under low power density excitation. Moreover, it is still challenging to generate small UCNPs, especially sub-10 nm, without sacrificing the upconversion efficiency. In addition, developing of commercially available systems of more versatile UCNPs with unique excitation and emission wavelength is critical to expand the applications of UCNPs. Meanwhile, further work is needed for better understanding the uptake, release rate, and toxicity of UCNPs for harnessing these nano-platforms for applications in photonics and biophotonics.

## References

[1] Auzel F. (2004). Upconversion and anti-stokes processes with f and d ions in solids. Chem. Rev..

[2] Wu X., Chen G.Y., Shen J., Li Z.J., Zhang Y.W., Han G. (2015). Upconversion Nanoparticles: A Versatile Solution to Multiscale Biological Imaging. Bioconjug. Chem..

[3] Wu S.W., Han G., Milliron D.J., Aloni S., Altoe V., Talapin D.V., Cohen B.E., Schuck P.J. (2009). Non-blinking and photostable upconverted luminescence from single lanthanide-doped nanocrystals. Proc. Natl. Acad. Sci. USA.

[4] Liu Q., Feng W., Yang T.S., Yi T., Li F.Y. (2013). Upconversion luminescence imaging of cells and small animals. Nat. Protoc..

[5] Chen G.Y., Shen J., Ohulchanskyy T.Y., Patel N.J., Kutikov A., Li Z.P., Song J., Pandey R.K., Agren H., Prasad P.N. (2012). (alpha-NaYbF_4_:Tm^3+^)/CaF_2_ Core/Shell Nanoparticles with Efficient Near-Infrared to Near-Infrared Upconversion for High-Contrast Deep Tissue Bioimaging. ACS Nano.

[6] Yang T.S., Liu Q., Li J.C., Pu S.Z., Yang P.Y., Li F.Y. (2014). Photoswitchable upconversion nanophosphors for small animal imaging *in vivo*. Rsc. Adv..

[7] Shen J., Zhao L., Han G. (2013). Lanthanide-doped upconverting luminescent nanoparticle platforms for optical imaging-guided drug delivery and therapy. Adv. Drug Deliver. Rev..

[8] Idris N.M., Jayakumar M.K.G., Bansal A., Zhang Y. (2015). Upconversion nanoparticles as versatile light nanotransducers for photoactivation applications. Chem. Soc. Rev..

[9] Wang J., Deng R.R., MacDonald M.A., Chen B.L., Yuan J.K., Wang F., Chi D.Z., Hor T.S.A., Zhang P., Liu G.K. (2014). Enhancing multiphoton upconversion through energy clustering at sublattice level. Nat. Mater..

[10] Liu Q., Sun Y., Yang T.S., Feng W., Li C.G., Li F.Y. (2011). Sub-10 nm Hexagonal Lanthanide-Doped NaLuF_4_ Upconversion Nanocrystals for Sensitive Bioimaging *in vivo*. J. Am. Chem. Soc..

[11] Ryu J., Park H.Y., Kim K., Kim H., Yoo J.H., Kang M., Im K., Grailhe R., Song R. (2010). Facile Synthesis of Ultrasmall and Hexagonal NaGdF_4_:Yb^3+^,Er^3+^ Nanoparticles with Magnetic and Upconversion Imaging Properties. J. Phys. Chem. C.

[12] Shen J., Chen G.Y., Vu A.M., Fan W., Bilsel O.S., Chang C.C., Han G. (2013). Engineering the Upconversion Nanoparticle Excitation Wavelength: Cascade Sensitization of Tri-doped Upconversion Colloidal Nanoparticles at 800 nm. Adv. Opt. Mater..

[13] Qiu P.Y., Zhou N., Chen H.Y., Zhang C.L., Gao G., Cui D.X. (2013). Recent advances in lanthanide-doped upconversion nanomaterials: Synthesis, nanostructures and surface modification. Nanoscale.

[14] Mai H.X., Zhang Y.W., Si R., Yan Z.G., Sun L.D., You L.P., Yan C.H. (2006). High-quality sodium rare-earth fluoride nanocrystals: Controlled synthesis and optical properties. J. Am. Chem. Soc..

[15] Yi G.S., Chow G.M. (2006). Synthesis of hexagonal-phase NaYF_4_:Yb,Er and NaYF4:Yb,Tm nanocrystals with efficient up-conversion fluorescence. Adv. Funct. Mater..

[16] Boyer J.C., Vetrone F., Cuccia L.A., Capobianco J.A. (2006). Synthesis of colloidal upconverting NaYF_4_ nanocrystals doped with Er^3+^, Yb^3+^ and Tm^3+^, Yb^3+^ via thermal decomposition of lanthanide trifluoroacetate precursors. J. Am. Chem. Soc..

[17] Boyer J.C., Cuccia L.A., Capobianco J.A. (2007). Synthesis of colloidal upconverting NaYF_4_: Er^3+^/Yb^3+^ and Tm^3+^/Yb^3+^ monodisperse nanocrystals. Nano Lett..

[18] Yi G.S., Chow G.M. (2007). Water-soluble NaYF_4_:Yb,Er(Tm)/NaYF_4_/polymer core/shell/shell nanoparticles with significant enhancement of upconversion fluorescence. Chem. Mater..

[19] Zeng J.H., Su J., Li Z.H., Yan R.X., Li Y.D. (2005). Synthesis and upconversion luminescence of hexagonal-phase NaYF_4_:Yb,Er, phosphors of controlled size and morphology. Adv. Mater..

[20] Wang X., Zhuang J., Peng Q., Li Y.D. (2005). A general strategy for nanocrystal synthesis. Nature.

[21] Liang X., Wang X., Zhuang J., Peng Q., Li Y.D. (2007). Synthesis of NaYF_4_ nanocrystals with predictable phase and shape. Adv. Funct. Mater..

[22] Wang Y., Tu L.P., Zhao J.W., Sun Y.J., Kong X.G., Zhang H. (2009). Upconversion Luminescence of β-NaYF4:Yb^3+^, Er^3+^@β-NaYF_4_ Core/Shell Nanoparticles: Excitation Power, Density and Surface Dependence. J. Phys. Chem. C.

[23] Wang F., Wang J.A., Liu X.G. (2010). Direct Evidence of a Surface Quenching Effect on Size-Dependent Luminescence of Upconversion Nanoparticles. Angew. Chem. Int. Ed..

[24] Shen J., Chen G.Y., Ohulchanskyy T.Y., Kesseli S.J., Buchholz S., Li Z.P., Prasad P.N., Han G. (2013). Tunable Near Infrared to Ultraviolet Upconversion Luminescence Enhancement in (α-NaYF4:Yb,Tm)/CaF_2_ Core/Shell Nanoparticles for *in situ* Real-time Recorded Biocompatible Photoactivation. Small.

[25] Zhong Y.T., Tian G., Gu Z.J., Yang Y.J., Gu L., Zhao Y.L., Ma Y., Yao J.N. (2014). Elimination of Photon Quenching by a Transition Layer to Fabricate a Quenching-Shield Sandwich Structure for 800 nm Excited Upconversion Luminescence of Nd^3+^ Sensitized Nanoparticles. Adv. Mater..

[26] Wu S., Butt H.J. (2015). Near-Infrared-Sensitive Materials Based on Upconverting Nanoparticles. Adv. Mater..

[27] Wen H.L., Zhu H., Chen X., Hung T.F., Wang B.L., Zhu G.Y., Yu S.F., Wang F. (2013). Upconverting Near-Infrared Light through Energy Management in Core-Shell-Shell Nanoparticles. Angew. Chem. Int. Ed..

[28] Lai J.P., Zhang Y.X., Pasquale N., Lee K.B. (2014). An Upconversion Nanoparticle with Orthogonal Emissions Using Dual NIR Excitations for Controlled Two-Way Photoswitching. Angew. Chem. Int. Ed..

[29] Zhao L., Kutikov A., Shen J., Duan C.Y., Song J., Han G. (2013). Stem Cell Labeling using Polyethylenimine Conjugated (α-NaYbF_4_:Tm^3+^)/CaF_2_ Upconversion Nanoparticles. Theranostics.

[30] Chen Z.G., Chen H.L., Hu H., Yu M.X., Li F.Y., Zhang Q., Zhou Z.G., Yi T., Huang C.H. (2008). Versatile synthesis strategy for carboxylic acid-functionalized upconverting nanophosphors as biological labels. J. Am. Chem. Soc..

[31] Zhang T.R., Ge J.P., Hu Y.X., Yin Y.D. (2007). A general approach for transferring hydrophobic nanocrystals into water. Nano Lett..

[32] Dong A.G., Ye X.C., Chen J., Kang Y.J., Gordon T., Kikkawa J.M., Murray C.B. (2011). A Generalized Ligand-Exchange Strategy Enabling Sequential Surface Functionalization of Colloidal Nanocrystals. J. Am. Chem. Soc..

[33] Inglefield D.L., Merritt T.R., Magill B.A., Long T.E., Khodaparast G.A. (2015). Upconverting nanocomposites dispersed in urea-containing acrylics. J. Mater. Chem. C.

[34] Min Y.Z., Li J.M., Liu F., Yeow E.K.L., Xing B.G. (2014). Near-Infrared Light-Mediated Photoactivation of a Platinum Antitumor Prodrug and Simultaneous Cellular Apoptosis Imaging by Upconversion-Luminescent Nanoparticles. Angew. Chem. Int. Ed..

[35] Xiong L.Q., Chen Z.G., Tian Q.W., Cao T.Y., Xu C.J., Li F.Y. (2009). High Contrast Upconversion Luminescence Targeted Imaging *in Vivo* Using Peptide-Labeled Nanophosphors. Anal. Chem..

[36] Yu X.F., Sun Z.B., Li M., Xiang Y., Wang Q.Q., Tang F.F., Wu Y.L., Cao Z.J., Li W.X. (2010). Neurotoxin-conjugated upconversion nanoprobes for direct visualization of tumors under near-infrared irradiation. Biomaterials.

[37] Priyam A., Idris M.N., Zhang Y. (2012). Gold nanoshell coated NaYF_4_ nanoparticles for simultaneously enhanced upconversion fluorescence and darkfield imaging. J. Mater. Chem..

[38] Hou Z.Y., Li C.X., Ma P.A., Li G.G., Cheng Z.Y., Peng C., Yang D.M., Yang P.P., Lin J. (2011). Electrospinning Preparation and Drug-Delivery Properties of an Up-conversion Luminescent Porous NaYF_4_:Yb^3+^, Er^3+^@Silica Fiber Nanocomposite. Adv. Funct. Mater..

[39] Xiang J., Xu L., Gong H., Zhu W., Wang C., Xu J., Feng L., Cheng L., Peng R., Liu Z. (2015). Antigen-Loaded Upconversion Nanoparticles for Dendritic Cell Stimulation, Tracking, and Vaccination in Dendritic Cell-Based Immunotherapy. ACS Nano.

[40] Yang Y.M., Velmurugan B., Liu X.G., Xing B.G. (2013). NIR Photoresponsive Crosslinked Upconverting Nanocarriers toward Selective Intracellular Drug Release. Small.

[41] Lai J.P., Shah B.R., Zhang Y.X., Yang L.T., Lee K.B. (2015). Real-Time Monitoring of ATP-Responsive Drug Release Using Mesoporous-Silica-Coated Multicolor Upconversion Nanoparticles. ACS Nano.

[42] Jiang S., Zhang Y. (2010). Upconversion Nanoparticle-Based FRET System for Study of siRNA in Live Cells. Langmuir.

[43] Guo H.C., Idris N.M., Zhang Y. (2011). LRET-Based Biodetection of DNA Release in Live Cells Using Surface-Modified Upconverting Fluorescent Nanoparticles. Langmuir.

[44] Fisher A.M.R., Murphree A.L., Gomer C.J. (1995). Clinical and Preclinical Photodynamic Therapy. Laser. Surg. Med..

[45] Idris N.M., Gnanasammandhan M.K., Zhang J., Ho P.C., Mahendran R., Zhang Y. (2012). *In vivo* photodynamic therapy using upconversion nanoparticles as remote-controlled nanotransducers. Nat. Med..

[46] Liu Y., Liu Y., Bu W., Cheng C., Zuo C., Xiao Q., Sun Y., Ni D., Zhang C., Liu J. (2015). Hypoxia Induced by Upconversion-Based Photodynamic Therapy: Towards Highly Effective Synergistic Bioreductive Therapy in Tumors. Angew. Chem. Int. Ed..

[47] Shan G.B., Weissleder R., Hilderbrand S.A. (2013). Upconverting Organic Dye Doped Core-Shell Nano-Composites for Dual-Modality NIR Imaging and Photo-Thermal Therapy. Theranostics.

[48] Guo Y.Y., Kumar M., Zhang P. (2007). Nanoparticle-based photosensitizers under CW infrared excitation. Chem. Mater..

[49] Shan J.N., Budijono S.J., Hu G.H., Yao N., Kang Y.B., Ju Y.G., Prud’homme R.K. (2011). Pegylated Composite Nanoparticles Containing Upconverting Phosphors and meso-Tetraphenyl porphine (TPP) for Photodynamic Therapy. Adv. Funct. Mater..

[50] Punjabi A., Wu X., Tokatli-Apollon A., El-Rifai M., Lee H., Zhang Y.W., Wang C., Liu Z., Chan E.M., Duan C.Y. (2014). Amplifying the red-emission of upconverting nanoparticles for biocompatible clinically used prodrug-induced photodynamic therapy. ACS Nano.

[51] Juzenas P., Juzeniene A., Kaalhus O., Iani V., Moan J. (2002). Noninvasive fluorescence excitation spectroscopy during application of 5-aminolevulinic acid *in vivo*. Photoch. Photobio. Sci..

[52] Chen Z.J., Sun W., Butt H.J., Wu S. (2015). Upconverting-Nanoparticle-Assisted Photochemistry Induced by Low-Intensity Near-Infrared Light: How Low Can We Go?. Chemistry.

[53] He S.Q., Krippes K., Ritz S., Chen Z.J., Best A., Butt H.J., Mailander V., Wu S. (2015). Ultralow-intensity near-infrared light induces drug delivery by upconverting nanoparticles. Chem. Commun..

[54] Chen Z.J., He S.Q., Butt H.J., Wu S. (2015). Photon Upconversion Lithography: Patterning of Biomaterials Using Near-Infrared Light. Adv. Mater..

[55] Lucky S.S., Idris N.M., Li Z.Q., Huang K., Soo K.C., Zhang Y. (2015). Titania Coated Upconversion Nanoparticles for Near-Infrared Light Triggered Photodynamic Therapy. ACS Nano.

[56] Hou Z.Y., Zhang Y.X., Deng K.R., Chen Y.Y., Li X.J., Deng X.R., Cheng Z.Y., Lian H.Z., Li C.X., Lin J. (2015). UV-Emitting Upconversion-Based TiO_2_ Photosensitizing Nanoplatform: Near-Infrared Light Mediated *in Vivo* Photodynamic Therapy via Mitochondria-Involved Apoptosis Pathway. ACS Nano.

[57] Saxton R.E., Paiva M.B., Lufkin R.B., Castro D.J. (1995). Laser Photochemotherapy—A Less Invasive Approach for Treatment of Cancer. Semin. Surg. Oncol..

[58] Dong B.A., Xu S., Sun J.A., Bi S., Li D., Bai X., Wang Y., Wang L.P., Song H.W. (2011). Multifunctional NaYF_4_:Yb^3+^,Er^3+^@Ag core/shell nanocomposites: Integration of upconversion imaging and photothermal therapy. J. Mater. Chem..

[59] Xiao Q.F., Zheng X.P., Bu W.B., Ge W.Q., Zhang S.J., Chen F., Xing H.Y., Ren Q.G., Fan W.P., Zhao K.L. (2013). A Core/Satellite Multifunctional Nanotheranostic for *in Vivo* Imaging and Tumor Eradication by Radiation/Photothermal Synergistic Therapy. J. Am. Chem. Soc..

